# Role of SIRT1 in Isoflurane Conditioning-Induced Neurovascular Protection against Delayed Cerebral Ischemia Secondary to Subarachnoid Hemorrhage

**DOI:** 10.3390/ijms22084291

**Published:** 2021-04-20

**Authors:** Meizi Liu, Keshav Jayaraman, Tusar Giri, Gregory J. Zipfel, Umeshkumar Athiraman

**Affiliations:** 1 Department of Anesthesiology, Washington University in Saint Louis, Saint Louis, MO 63110, USA; meizi.l@wustl.edu (M.L.); keshav.jayaraman@wustl.edu (K.J.); tusargiri@wustl.edu (T.G.); 2 Department of Neurological Surgery, Washington University in Saint Louis, Saint Louis, MO 63110, USA; zipfelg@wustl.edu

**Keywords:** isoflurane conditioning, aneurysmal subarachnoid hemorrhage, delayed cerebral ischemia, SIRT1

## Abstract

We recently reported that isoflurane conditioning provided multifaceted protection against subarachnoid hemorrhage (SAH)-induced delayed cerebral ischemia (DCI), and this protection was through the upregulation of endothelial nitric oxide synthase (eNOS). SIRT1, an NAD-dependent deacetylase, was shown to be one of the critical regulators of eNOS. The aim of our current study is to examine the role of SIRT1 in isoflurane conditioning-induced neurovascular protection against SAH-induced DCI. Mice were divided into four groups: sham, SAH, or SAH with isoflurane conditioning (with and without EX-527). Experimental SAH via endovascular perforation was performed. Anesthetic conditioning was performed with isoflurane 2% for 1 h, 1 h after SAH. EX-527, a selective SIRT1 inhibitor, 10 mg/kg was injected intraperitoneally immediately after SAH in the EX-527 group. SIRT1 mRNA expression and activity levels were measured. Vasospasm, microvessel thrombosis, and neurological outcome were assessed. SIRT1 mRNA expression was downregulated, and no difference in SIRT1 activity was noted after isoflurane exposure. Isoflurane conditioning with and without EX-527 attenuated vasospasm, microvessel thrombosis and improved neurological outcomes. Our data validate our previous findings that isoflurane conditioning provides strong protection against both the macro and micro vascular deficits induced by SAH, but this protection is likely not mediated through the SIRT1 pathway.

## 1. Introduction

Aneurysmal subarachnoid hemorrhage (SAH) is a severe form of stroke with extremely high morbidity and mortality [1], much of which stems from a secondary brain injury process called delayed cerebral ischemia (DCI) [2]. For years, DCI was solely attributed to vasospasm, which is characterized by large artery narrowing 4–12 d after SAH [3]. However, in recent years, several additional processes affecting the microcirculation have been causally linked to DCI, including autoregulatory dysfunction [4,5] and microvessel thrombosis [6,7]. Several therapies have been tried and failed to treat DCI, which is likely due to targeting individual elements of what has proven to be a multifactorial process. So, future therapies should be designed to target both large artery vasospasm and microcirculatory deficits to be effective. To address this issue, we and others have applied a therapeutic strategy—*conditioning*—that leverages endogenous molecular mechanisms to exert a powerful and remarkably pleiotropic protective effect against DCI after SAH. For years, neurons were felt to be the principal target of this response (neuronal conditioning) [8], but multiple lines of evidence now show that glia (glial conditioning) [9] and vessels (vascular conditioning) [10,11,12,13,14,15] are also causally involved. The latter is of special interest for SAH, given the central role vascular deficits (vasospasm, autoregulatory dysfunction, and microvessel thrombi) play in DCI [3,4,5,6,7]. This vascular protection—along with the 4–12 d window between SAH and DCI—makes SAH an ideal target for the conditioning strategies to maximize the neurovascular protection.

SIRT1 (silent mating type information regulation 2 homolog), a known nicotinamide adenine dinucleotide (NAD)-dependent deacetylase, is present ubiquitously in the brain [16,17], and several studies have demonstrated a protective role of SIRT1 in various neurological disorders [18,19,20] including SAH [21,22]. Recently, Vellimana et al. [22] provided critical evidence showing that SIRT1 is a key mediator of *hypoxic* conditioning-induced protection against SAH-induced DCI. SIRT1 is a well-known regulator of numerous molecular pathways, several of which have been linked to the pathophysiology of DCI including endothelial nitric oxide synthase (eNOS) [23], hypoxia inducible factor 1α (HIF-1α) [24], and others [25,26,27,28]. In fact, work from the same laboratory has also causally linked the DCI protection afforded by hypoxic conditioning to eNOS [29], which suggests that the SIRT1-eNOS cascade is essential to the observed DCI protection. Interestingly, *isoflurane* conditioning has also been shown to provide strong DCI protection in SAH, and to do so via upregulation of eNOS [15] and HIF-1α [14]. Therefore, it is logical to posit that anesthetic conditioning-induced DCI protection after SAH may also be critically dependent on SIRT1. Hence, our current study is focused on investigating the role of SIRT1 in isoflurane conditioning-induced neurovascular protection against SAH.

## 2. Results

### 2.1. SIRT1 mRNA Expression and Activity after Isoflurane Conditioning in Naive Animals

**Figure 1:**
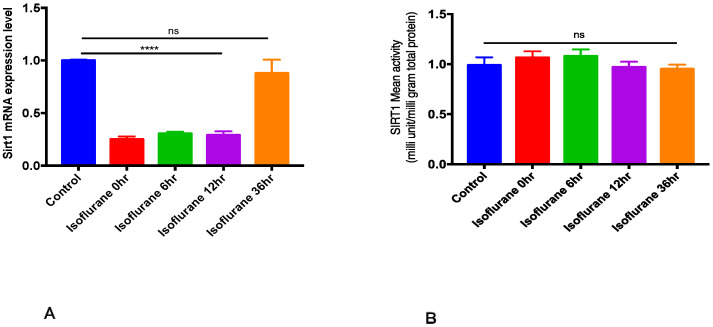
Isoflurane and SIRT1 mRNA expression and activity. Wild-type mice were exposed to air or 2% isoflurane for 1 h and cortical tissue was harvested immediately and at 6, 12, and 36 h after isoflurane exposure were subjected to RT-PCR and ELISA. Data indicate mean ± SEM. (**A**) **** *p* < 0.0001 vs. control (no isoflurane) by ANOVA, ns—nonsignificant. (**B**) ns—nonsignificant, *p* > 0.05 vs. control (no isoflurane) by ANOVA. (*n* = 5).

SIRT1 mRNA expression and activity were measured at different time points after single time exposure to 2% isoflurane for one hour. Isoflurane conditioning significantly decreased SIRT1 mRNA expression until 12 h (*p* < 0.05), and a nonsignificant decrease in the SIRT1 mRNA expression was noted at 36 h (Figure 1A). No significant difference in SIRT1 activity was noted between the control and isoflurane exposed groups (*p* > 0.05, Figure 1B).

### 2.2. Isoflurane Conditioning with or without EX-527 Administration Attenuated SAH-Induced Large Artery Vasospasm and Improved Neurological Deficits in Wild Type Mice

**Figure 2:**
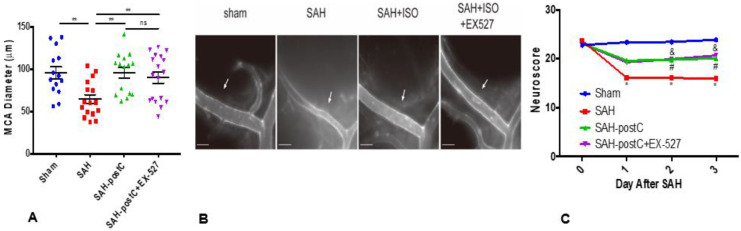
Isoflurane conditioning attenuates SAH-induced vasospasm and improves neurological outcomes with and without EX-527: Wild-type mice were subjected to sham surgery or endovascular perforation SAH and treated with vehicle (DMSO+1.2% cyclodextrin+PBS1X) or EX-527 (10mg/kg) diluted with vehicle. Two out of four groups were exposed to 2% isoflurane for one hour (postC), one hour after SAH, and one isoflurane group out of two received a single dose of EX-527 immediately after SAH. Vasospasm (**A**) was assessed on Day 3. Neuroscore was assessed baseline and daily for 3 days (**C**). Data indicate mean ± SEM. (**A**) ** *p* < 0.01 sham vs. SAH, ** *p* < 0.01 SAH vs. SAH+Isoflurane, SAH+Isoflurane+EX-527, *p* > 0.05 SAH+Isoflurane vs. SAH+Isoflurane+EX-527, by ANOVA and Newman–Keuls multiple comparison test. ns—nonsignificant. (**B**) Representative images for vasospasm. ISO—Isoflurane. Arrow mark represents the middle cerebral artery vessel. Scale bar = 500 μm. (**C**) *p* < 0.05 Sham vs. SAH, # *p* < 0.05 SAH vs. SAH-postC, & *p* < 0.05 SAH vs. SAH-postC+EX-527 by ANOVA and two-way repeated measures ANOVA with Newman–Keuls multiple comparisons test.

Out of a total 67 wild-type mice used in these experiments, four animals died in the SAH group and none died in the sham group. Animals subjected to SAH surgery were found to have SAH at the ventral surface of the brain at the time of animal sacrifice, but none in sham group were noted to have SAH. Significant vasospasm and neurological deficits were noted in the mice subjected to SAH as compared to sham (*p* < 0.05, Figure 2A,C). Isoflurane conditioning afforded protection against vasospasm and neurological deficits caused by SAH. The administration of EX-527, a selective Sirt1 inhibitor, did not block isoflurane’s effect on SAH-induced vasospasm and neurological deficits (*p* < 0.05, Figure 2A,C).

### 2.3. Isoflurane Conditioning with or without EX-527 Administration Attenuated SAH-Induced Microvessel Thrombosis in Wild-Type Mice

**Figure 3:**
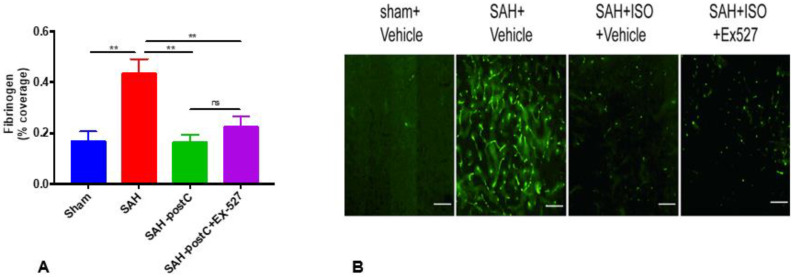
Isoflurane conditioning attenuates SAH-induced microvessel thrombosis with and without EX-527. Wild-type mice were subjected to sham surgery or endovascular perforation SAH and treated with vehicle (DMSO+1.2% cyclodextrin+PBS1X) or EX-527 (10mg/kg) diluted with vehicle. Two out of four groups were exposed to 2% isoflurane for one hour (postC), one hour after SAH, and one isoflurane group out of two received a single dose of EX-527 immediately after SAH. Microvessel thrombosis was assessed on day 3. Data indicate mean±SEM. (**A**) ** *p* < 0.01 sham vs. SAH, ** *p* < 0.01 SAH vs. SAH-postC, SAH-postC+EX-527, *p* > 0.05 SAH-postC, SAH-postC+EX-527, by ANOVA and Newman–Keuls multiple comparisons test. (*n =* 5) ns—nonsignificant. (**B**) Representative images for microvessel thrombosis. ISO—Isoflurane. Scale bar = 100 μm.

Next, we investigated if isoflurane conditioning attenuated microvessel thrombosis induced by SAH and if administration of EX-527 blocked isoflurane’s protective effect against SAH-induced microvessel thrombosis. SAH induced significant microvessel thrombosis, and isoflurane conditioning significantly attenuated microvessel thrombosis (*p* < 0.05, Figure 3A). The administration of EX-527 did not eliminate isoflurane’s protection against SAH-induced microvessel thrombosis (Figure 3A).

## 3. Discussion

The main findings in our study are as follows: (1) Isoflurane conditioning provides robust protection against the macro- and micro-vascular components of SAH-induced DCI (vasospasm and microvessel thrombosis), leading to improved neurological deficits; (2) Isoflurane conditioning does not increase SIRT1 expression or activity; and (3) Administration of the SIRT1-specific inhibitor, EX-527, did not eliminate isoflurane-induced protection against DCI. These finding provide strong evidence that the robust protective effect of isoflurane conditioning on DCI and neurological deficits are not mediated via SIRT1. This finding is important for two main reasons: (1) It demonstrates that the mechanisms associated with conditioning-induced DCI protection vary according to the agent employed (e.g., isoflurane vs. hypoxia); and (2) It indicates that upstream molecules other than SIRT1 are primarily responsible for the impact of isoflurane conditioning on DCI protection.

SIRT1 is a well-known NAD-dependent epigenetic modulator that regulates gene transcription by deacetylating histones and other transcription regulators impacting numerous functions including inflammation, vessel function, and thrombosis [30,31]. Several studies have implicated a protective role of SIRT1 against various neurological disorders including cerebral ischemia [32,33]. With respect to SAH-induced brain injury, SIRT1 has been linked to two of the most important forms of post-ictal secondary brain injury: EBI [34,35,36] and DCI [22]. In the case of anesthetic conditioning, there is a growing body of preclinical and clinical evidence indicating a strong protective effect of inhalational anesthetics against SAH-induced DCI [15,37,38,39,40]. Milner et al. [14] previously noted that isoflurane conditioning provides robust protection against multiple elements of DCI including improved neurologic outcomes in a mouse model of SAH. This protection was casually linked to endothelial cell-derived HIF-1α [14]. More recently, we have shown that isoflurane conditioning provides strong protection against vasospasm-induced DCI and neurological deficits in a mouse model of SAH [15,39] and that this protection is critically dependent on eNOS [15].

Available evidence suggests that SIRT1 plays a fundamental role in regulating endothelial nitric oxide and maintaining the endothelium dependent vascular tone [23]. It is also shown that SIRT1 is necessary for stabilizing HIF-1α [24]. Interestingly, a mechanistic link has been suggested between these molecules (SIRT1, HIF-1α, and eNOS) [23,24,41,42,43,44], although the role of SIRT1 on anesthetic conditioning-induced DCI protection in SAH has yet to be examined.

Our data show that brief exposure to 2% isoflurane significantly downregulated SIRT1 gene expression but had no significant impact on SIRT1 activity. These results are consistent with past reports showing that isoflurane significantly downregulates SIRT1 gene and protein expression in mice [45,46]. Similar results have been reported with another inhalational anesthetic, sevoflurane [47,48].

Our data also show that brief exposure to 2% isoflurane beginning one hour after SAH induction protects against one key element of DCI—large artery vasospasm, which is consistent with our past results [15]. In addition, we show that isoflurane conditioning protects against another critical component of DCI—microvessel thrombosis. These data provide strong evidence that isoflurane conditioning induces robust protection against both macro- *and* micro-vessel components of DCI, leading to improved short-term neurological outcome. A therapeutic strategy that can attenuate both macro- and micro-vessel dysfunction has great translational potential for SAH patients.

Next, we examined if administration of a highly selective SIRT1 inhibitor, EX-527 [49], abolishes the DCI protection afforded by isoflurane conditioning. Interestingly, the administration of EX-527 did not eliminate isoflurane conditioning-induced protection against vasospasm, microvessel thrombosis, or neurological deficits. These results, along with our observations regarding the impact of isoflurane on SIRT1 gene expression and activity, indicate that isoflurane-induced DCI protection in SAH is not dependent on SIRT1.

In total, our findings indicate that isoflurane conditioning-induced protection against vasospasm and microvessel thrombosis after SAH is not mediated through SIRT1. Understanding the molecular underpinnings of anesthetic conditioning-based neurovascular protection in SAH is critical, as this strategy has a great translational potential given that isoflurane and other commonly used anesthetic agents are already approved by the FDA for use in patients and have shown to possess excellent safety profile.

Limitations of the study: Our study has several limitations. (1) SAH in our experiments were confirmed by the occurrence of apnea after perforation and by the presence of blood during the animal sacrifice. However, we did not quantify the severity of SAH, and it is possible that the severity of SAH in the animals were not homogenously distributed. (2) Though EX-527 is a highly specific SIRT1 inhibitor, it is possible that other SIRT1 inhibitors may have different effects on isoflurane conditioning in SAH-induced DCI. (3) While we show that Sirt1 may not be involved in isoflurane conditioning induced DCI protection, the mechanism behind its protection is not explored in the current study.

## 4. Materials and Methods

### 4.1. Experimental Animals

**Figure 4:**
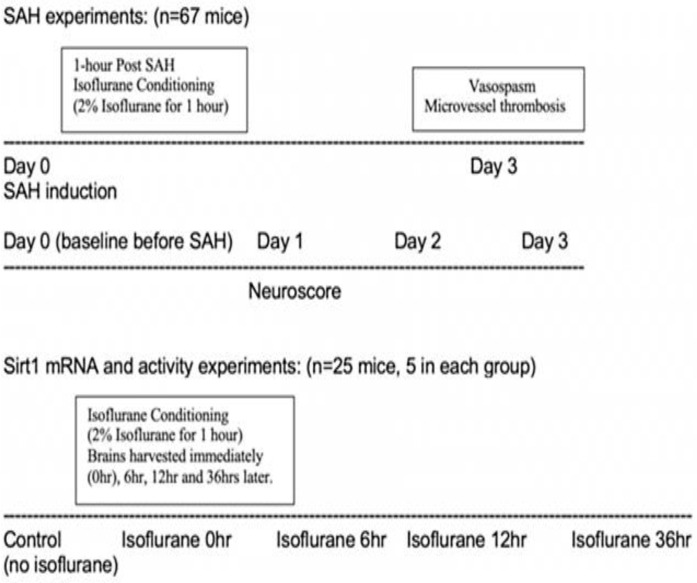
Flow chart representing the overall experimental design. SAH—subarachnoid hemorrhage. Sirt1—silent mating type information regulation 2 homolog.

Experiments in the study were approved by Washington University in Saint Louis animal care and use committee (protocol no. 20180080, Approval date: 07/22/2019). C57BL/6J male mice (12 weeks old) were acquired from Jackson laboratories (Bar Harbor, ME) for the experiments. Experiments were conducted in a randomized and blinded manner. Mice underwent sham or SAH surgery and were divided into the following groups, sham (*n* = 14), SAH (*n* = 16), SAH with isoflurane conditioning (*n* = 19), and SAH with isoflurane conditioning and EX-527 (*n* = 18). A separate cohort of mice was utilized for examining Sirt1 mRNA and activity levels after isoflurane conditioning. A total of 25 wild-type mice with *n* = 5 in each group have been utilized for these experiments. Figure 4 shows the overall experimental design of the study.

### 4.2. Experimental SAH

Endovascular perforation SAH was performed per our published methods [14,15]. Briefly, mice were anesthetized with isoflurane (4% induction, 1.5% maintenance) in room air with a core body temperature maintained at 37 °C. A midline incision was made in the neck, the external carotid artery (ECA) was exposed, and a 5-0 nylon suture was introduced through it and advanced distally through the internal carotid artery (ICA) until reaching the ICA bifurcation. The suture was advanced further to induce SAH and then removed, and the ECA was ligated. Mice undergoing sham operation underwent the same steps except for perforation. Post-surgery, mice were returned to their cages when fully recovered.

### 4.3. Isoflurane Conditioning

Mice were administered 2% isoflurane in room air for one hour, beginning one-hour post SAH surgery. Sham mice were administered room air for one hour without isoflurane. Isoflurane concentration was monitored using a gas analyzer (Datex Ohmeda, Capnomac Ultima, Louisville, KY, USA). A normal body temperature at 37 °C was maintained utilizing the homeothermic blanket.

### 4.4. EX-527 (a Selective SIRT1 Inhibitor) Treatment

To examine the role of SIRT1 in isoflurane conditioning-induced neurovascular protection, a separate cohort of animals were injected intraperitoneally with EX-527 (10 mg/kg, Millipore Sigma, Saint Louis, MO, USA) diluted with DMSO+1.2% cyclodextrin+PBS1X immediately after SAH induction. Another three groups received equal amounts of DMSO+1.2% cyclodextrin+PBS1X intraperitoneally without EX-527 as the control solution.

### 4.5. Vasospasm Assessment

Vasospasm assessment was performed per our published methods using ROX SE, 72 h after SAH surgery. [50] Briefly, mice were anesthetized, transcardially perfused with PBS 1×, 10% formalin, and ROX-SE. Blood vessels in the circle of Willis were imaged under a fluorescent microscope using a CCD camera (Cool SNAP EZ, Photometrics, Tucson, AZ, USA) and MetaMorph^®^ software (Universal Imaging, West Chester, PA, USA). Vasospasm measurement for each brain sample was obtained by recording the narrowest diameter within the first 1000 μm segment of the left (ipsilateral) MCA.

### 4.6. Neurobehavioral Assessment

According to our previously published protocol [14,15], neurobehavioral outcome was examined baseline and for next three days after SAH. Briefly, neurological function was graded based on a motor score (0–12) and a sensory score (4–12) that assessed spontaneous activity, symmetry of limb movements, climbing, balance and coordination body proprioception and vibrissa, visual, and tactile responses.

### 4.7. Microvessel Thrombosis Assessment

Cortical microvessel thrombosis was assessed by immunofluorescence staining for fibrinogen. Briefly, brain samples fixed in 4% paraformaldehyde were sectioned into 50 μm coronal sections. Brain sections were incubated with a blocking buffer (0.1% Triton X-100 TBS-T with 0.2% milk and 1% BSA) and then incubated with rabbit anti-fibrinogen antibody (1:3000, Abcam, Cambridge, MA, USA) overnight at 4 °C, followed by incubation with donkey anti-rabbit secondary antibody (1:2000, Invitrogen Waltham, MA, USA), overnight at 4 °C Sections mounted on the slide were analyzed for the fibrinogen staining under NanoZoomer 2.0-HT digital slide scanner (Hamamatsu Corporation, Bridgewater, NJ, USA). The microvessel thrombosis density was calculated with Image J.

### 4.8. Quantitative Polymerase Chain Reaction (qPCR) for SIRT1 Gene Expression

Isoflurane-induced changes in the SIRT1 mRNA expression were measured by TaqMan^®^ qPCR assay. Total RNA was prepared from cerebral cortex using RNeasy mini kit (Qiagen) and converted to cDNA using a SuperScript^®^ IV VILO^™^ master mix kit (Invitrogen) according to the manufacturer’s instructions. Template cDNA (25 ng) was combined with a ready-to-use TaqMan Fast Advanced qPCR Master Mix (ThermoFisher Scientific, Waltham, MA, USA) for gene expression experiments with a custom TaqMan^®^ Sirt1 probe. Thermal cycling was performed in 7500 Fast Real-Time PCR System (Applied Biosystems^®^, Foster City, CA, USA), and the threshold cycle (Ct) values for all samples were calculated using proprietary software. Relative mRNA expression, normalized to the geometric mean of 2 reference genes (gapdh and actb), was calculated using the 2^−ΔΔCT^ method with sex-matched control samples as reference.

### 4.9. Enzyme-Linked Immunosorbent Assay (ELISA) for SIRT1 Protein Expression

Protein lysates were prepared from approximately 60 mg of cerebral cortex, and protein concentrations were determined using BCA Protein Assay Kit (ThermoFisher Scientific Inc., Waltham, MA, USA), and stored at −80 °C freezer until further use. SIRT1 activity was measured using a Sirtuin Activity Assay Kit (catalog # K324-100, Bio Vision Inc., Milpitas, CA, USA) according to the manufacturer’s instructions. All samples were performed in duplicate, and fluorescence intensity was read at Ex 400 nm/Em 505 in end point mode with Tecan Infinite^®^ M200 PRO multimode plate reader. Results calculated from the standard curve for SIRT1 activity were expressed as mU/mg total brain protein.

### 4.10. Statistical Analysis

Power analysis for individual experiments were calculated based on our pilot studies and previous published studies. For Sirt1 mRNA levels and protein activity, we estimate 80% power to detect 20% difference between groups with an *n* = 5 per group based on a one-way ANOVA model at a significance level of 5%. When comparing vasospasm and neurological outcome, we estimate 80% power to detect 50% difference between groups with *n* = 14–18 per group based on a one-way ANOVA model at a significance level of 5%. For microvessel thrombosis, we estimate 80% power to detect 25% difference between groups with *n* = 5–8 per group, based on two-way ANOVA models without interaction at a significance level of 5%. Data are represented as the mean ± SEM. ANOVA with Newman–Keuls multiple comparison test was utilized to analyze large artery vasospasm, microvessel thrombosis, SIRT1 gene expression, and protein activity. Two-way repeated measures ANOVA followed by a Newman–Keuls multiple comparison test was used to analyze neurological outcome. *p* < 0.05 was considered statistically significant.

## Data Availability

All data are present within the manuscript or available by request to corresponding author, Umeshkumar Athiraman (uathira@wustl.edu).

## Abbreviations

SAH	Subarachnoid hemorrhage
DCI	Delayed Cerebral Ischemia
SIRT1	Silent mating type information regulation 2 homolog
NAD	Nicotinamide adenine dinucleotide
eNOS	Endothelial nitric oxide synthase
HIF-1α	Hypoxia inducible factor 1α
MCA	Middle cerebral artery
ICA	Internal carotid artery
ECA	External Carotid artery
ROX-SE	5-(and-6)-Carboxy-X-rhodamine, succinimidyl ester
PBS	Phosphate-buffered saline
DMSO	Dimethyl Sulfoxide
TBS-T	Tris-Buffered Saline + Tween
BSA	Bovine Serum Albumin
Gapdh	Glyceraldehyde 3-phosphate dehydrogenase
ActB	Actin Beta

## References

[1] Broderick J.P., Brott T.G., Duldner J.E., Tomsick T., Leach A. (1994). Initial and recurrent bleeding are the major causes of death following subarachnoid hemorrhage. Stroke.

[2] Brathwaite S., Macdonald R.L. (2014). Current Management of Delayed Cerebral Ischemia: Update from Results of Recent Clinical Trials. Transl. Stroke Res..

[3] van Gijn J., Kerr R.S., Rinkel G.J. (2007). Subarachnoid haemorrhage. Lancet.

[4] Dernbach P.D., Little J.R., Jones S.C., Ebrahim Z.Y. (1988). Altered Cerebral Autoregulation and CO2 Reactivity after Aneurysmal Subarachnoid Hemorrhage. Neurosurgery.

[5] Touho H., Ueda H. (1994). Disturbance of autoregulation in patients with ruptured intracranial aneurysms: Mechanism of cortical and motor dysfunction. Surg. Neurol..

[6] Sabri M., Ai J., Lakovic K., D’Abbondanza J., Ilodigwe D., Macdonald R. (2012). Mechanisms of microthrombi formation after experimental subarachnoid hemorrhage. Neuroscience.

[7] Vergouwen M.D.I., Vermeulen M., Coert B.A., Stroes E.S.G., Roos Y.B.W.E.M. (2008). Microthrombosis after Aneurysmal Subarachnoid Hemorrhage: An Additional Explanation for Delayed Cerebral Ischemia. Br. J. Pharmacol..

[8] Gidday J.M. (2006). Cerebral preconditioning and ischaemic tolerance. Nat. Rev. Neurosci..

[9] Trendelenburg G., Dirnagl U. (2005). Neuroprotective role of astrocytes in cerebral ischemia: Focus on ischemic preconditioning. Glia.

[10] Bastide M., Gelé P., Pétrault O., Pu Q., Caliez A., Robin E., Deplanque D., Duriez P., Bordet R. (2003). Delayed Cerebrovascular Protective Effect of Lipopolysaccharide in Parallel to Brain Ischemic Tolerance. Br. J. Pharmacol..

[11] Vlasov T.D., Korzhevskii D.E., Polyakova E.A. (2005). Ischemic Preconditioning of the Rat Brain as a Method of Endothelial Protection from Ischemic/Repercussion Injury. Neurosci. Behav. Physiol..

[12] Masada T., Hua Y., Xi G., Ennis S.R., Keep R.F. (2001). Attenuation of Ischemic Brain EDEMA and Cerebrovascular Injury after Ischemic Preconditioning in the Rat. Br. J. Pharmacol..

[13] Stowe A.M., Altay T., Bs A.B.F., Gidday J.M. (2011). Repetitive hypoxia extends endogenous neurovascular protection for stroke. Ann. Neurol..

[14] Milner E., Johnson A.W., Nelson J.W., Harries M.D., Gidday J.M., Han B.H., Zipfel G.J. (2015). HIF-1α Mediates Isoflurane-Induced Vascular Protection in Subarachnoid Hemorrhage. Ann. Clin. Transl. Neurol..

[15] Athiraman U., Jayaraman K., Liu M., Giri T., Yuan J., Zipfel G.J. (2020). Role of Endothelial Nitric Oxide Synthase in Isoflurane Conditioning-Induced Neurovascular Protection in Subarachnoid Hemorrhage. J. Am. Heart Assoc..

[16] Ramadori G., Lee C.E., Bookout A.L., Lee S., Williams K.W., Anderson J., Elmquist J.K., Coppari R. (2008). Brain SIRT1: Anatomical Distribution and Regulation by Energy Availability. J. Neurosci..

[17] Herskovits A.Z., Guarente L. (2014). SIRT1 in Neurodevelopment and Brain Senescence. Neuron.

[18] Pallàs M., Pizarro J., Gutierrez-Cuesta J., Crespo-Biel N., Alvira D., Tajes M., Yeste-Velasco M., Folch J., Canudas A., Sureda F. (2008). Modulation of SIRT1 expression in different neurodegenerative models and human pathologies. Neuroscience.

[19] Tang B.L. (2009). Sirt1′s Complex Roles in Neuroprotection. Cell. Mol. Neurobiol..

[20] Zhang F., Wang S., Gan L., Vosler P.S., Gao Y., Zigmond M.J., Chen J. (2011). Protective effects and mechanisms of sirtuins in the nervous system. Prog. Neurobiol..

[21] Vellimana A.K., Diwan D., Clarke J., Gidday J.M., Zipfel G.J. (2018). SIRT1 Activation: A Potential Strategy for Harnessing Endogenous Protection Against Delayed Cerebral Ischemia After Subarachnoid Hemorrhage. Neurosurgery.

[22] Vellimana A.K., Aum D.J., Diwan D., Clarke J.V., Nelson J.W., Lawrence M., Han B.H., Gidday J.M., Zipfel G.J. (2020). SIRT1 mediates hypoxic preconditioning induced attenuation of neurovascular dysfunction following subarachnoid hemorrhage. Exp. Neurol..

[23] Mattagajasingh I., Kim C.-S., Naqvi A., Yamamori T., Hoffman T.A., Jung S.-B., DeRicco J., Kasuno K., Irani K. (2007). SIRT1 promotes endothelium-dependent vascular relaxation by activating endothelial nitric oxide synthase. Proc. Natl. Acad. Sci. USA.

[24] Rane S., He M., Sayed D., Vashistha H., Malhotra A., Sadoshima J., Vatner D.E., Vatner S.F., Abdellatif M. (2009). Downregulation of MiR-199a Derepresses Hypoxia-Inducible Factor-1α and Sirtuin 1 and Recapitulates Hypoxia Preconditioning in Cardiac Myocytes. Circ. Res..

[25] Rajamohan S.B., Pillai V.B., Gupta M., Sundaresan N.R., Birukov K.G., Samant S., Hottiger M.O., Gupta M.P. (2009). SIRT1 Promotes Cell Survival under Stress by Deacetylation-Dependent Deactivation of Poly(ADP-Ribose) Polymerase 1. Mol. Cell. Biol..

[26] Lee J.S., Park K.Y., Min H.G., Lee S.J., Kim J., Choi J., Kim W., Cha H. (2010). Negative regulation of stress-induced matrix metallopro-teinase-9 by Sirt1 in skin tissue. Exp. Dermatol..

[27] Breitenstein A., Stein S., Holy E.W., Camici G.G., Lohmann C., Akhmedov A., Spescha R., Elliott P.J., Westphal C.H., Matter C.M. (2010). Sirt1 inhibition promotes in vivo arterial thrombosis and tissue factor expression in stimulated cells. Cardiovasc. Res..

[28] Chaitanya G.V., Alexander J.S., Babu P.P. (2010). PARP-1 cleavage fragments: Signatures of cell-death proteases in neurodegeneration. Cell Commun. Signal..

[29] Vellimana A.K., Milner E., Azad T.D., Harries M.D., Zhou M.-L., Gidday J.M., Han B.H., Zipfel G.J. (2011). Endothelial Nitric Oxide Synthase Mediates Endogenous Protection Against Subarachnoid Hemorrhage-Induced Cerebral Vasospasm. Stroke.

[30] Pillarisetti S. (2008). A Review of Sirt1 and Sirt1 Modulators in Cardiovascular and Metabolic Diseases. Recent Pat. Cardiovasc. Drug Discov..

[31] Satoh A., Stein L., Imai S. (2011). The Role of Mammalian Sirtuins in the Regulation of Metabolism, Aging, and Longevity. Organotypic Models Drug Dev..

[32] Raval A.P., Dave K.R., Perez-Pinzon M.A. (2005). Resveratrol Mimics Ischemic Preconditioning in the Brain. Br. J. Pharmacol..

[33] Perez-Pinzon M.A., Koronowski K.B. (2015). Sirt1 in cerebral ischemia. Brain Circ..

[34] Zhang X.-S., Wu Q., Wu L.-Y., Ye Z.-N., Jiang T.-W., Ling-Yun W., Zhuang Z., Zhou M.-L., Zhang X., Hang C.-H. (2016). Sirtuin 1 activation protects against early brain injury after experimental subarachnoid hemorrhage in rats. Cell Death Dis..

[35] Qian C., Jin J., Chen J., Li J., Yu X., Mo H., Chen G. (2017). SIRT1 activation by resveratrol reduces brain edema and neuronal apoptosis in an experimental rat subarachnoid hemorrhage model. Mol. Med. Rep..

[36] Li Z., Han X. (2018). Resveratrol alleviates early brain injury following subarachnoid hemorrhage: Possible involvement of the AMPK/SIRT1/autophagy signaling pathway. Biol. Chem..

[37] Athiraman U., Aum D., Vellimana A.K., Osbun J.W., Dhar R., Tempelhoff R., Zipfel G.J. (2020). Evidence for a conditioning effect of inhalational anesthetics on angiographic vasospasm after aneurysmal subarachnoid hemorrhage. J. Neurosurg..

[38] Athiraman U., Dhar R., Jayaraman K., Karanikolas M., Helsten D., Yuan J., Lele A.V., Rath G.P., Tempelhoff R., Roth S. (2020). Conditioning Effect of Inhalational Anesthetics on Delayed Cerebral Ischemia After Aneurysmal Subarachnoid Hemorrhage. Neurosurgery.

[39] Athiraman U., Liu M., Jayaraman K., Yuan J., Mehla J., Zipfel G.J. (2021). Anesthetic and subanesthetic doses of isoflurane conditioning provides strong protection against delayed cerebral ischemia in a mouse model of subarachnoid hemorrhage. Brain Res..

[40] Athiraman U., Zipfel G.J. (2020). Anesthetic Conditioning for Secondary Brain Injury After Aneurysmal Subarachnoid Hemorrhage. World Neurosurg..

[41] Rodriguez-Miguelez P., Lima-Cabello E., Martínez-Flórez S., Almar M., Cuevas M.J., González-Gallego J. (2015). Hypoxia-inducible factor-1 modulates the expression of vascular endothelial growth factor and endothelial nitric oxide synthase induced by eccentric exercise. J. Appl. Physiol..

[42] Alique M., Sánchez-López E., Bodega G., Giannarelli C., Carracedo J., Ramírez R. (2020). Hypoxia-Inducible Factor-1α: The Master Regulator of Endothelial Cell Senescence in Vascular Aging. Cells.

[43] Nagel S., Papadakis M., Chen R., Hoyte L.C., Brooks K.J., Gallichan D., Sibson N.R., Pugh C., Buchan A.M. (2010). Neuroprotection by Dimethyloxalylglycine following Permanent and Transient Focal Cerebral Ischemia in Rats. Br. J. Pharmacol..

[44] Mi D.-H., Fang H.-J., Zheng G.-H., Liang X.-H., Ding Y.-R., Liu X., Liu L.-P. (2018). DPP-4 inhibitors promote proliferation and migration of rat brain microvascular endothelial cells under hypoxic/high-glucose conditions, potentially through the SIRT1/HIF-1/VEGF pathway. CNS Neurosci. Ther..

[45] Hong-Qiang H., Mang-Qiao S., Fen X., Shan-Shan L., Hui-Juan C., Wu-Gang H., Wen-Jun Y., Zheng-Wu P. (2018). Sirt1 mediates improvement of isoflurane-induced memory impairment following hyperbaric oxygen preconditioning in middle-aged mice. Physiol. Behav..

[46] Fang X., Han Q., Li S., Zhao Y., Luo A. (2017). Chikusetsu saponin IVa attenuates isoflurane-induced neurotoxicity and cognitive def-icits via SIRT1/ERK1/2 in developmental rats. Am. J. Transl. Res..

[47] Liu L., Liu C., Fang L. (2020). AMPK-SIRT1 pathway dysfunction contributes to neuron apoptosis and cognitive impairment induced by sevoflurane. Mol. Med. Rep..

[48] Yang X.-Y., Li Q.-J., Zhang W.-C., Zheng S.-Q., Qu Z.-J., Xi Y., Wang G. (2020). AMPK-SIRT1-PGC1α Signal Pathway Influences the Cognitive Function of Aged Rats in Sevoflurane-Induced Anesthesia. J. Mol. Neurosci..

[49] Peck B., Chen C.-Y., Ho K.-K., Di Fruscia P., Myatt S.S., Coombes R.C., Fuchter M.J., Hsiao C.-D., Lam E.W.-F. (2010). SIRT Inhibitors Induce Cell Death and p53 Acetylation through Targeting Both SIRT1 and SIRT2. Mol. Cancer Ther..

[50] Aum D.J., Vellimana A.K., Singh I., Milner E., Nelson J.W., Han B.H., Zipfel G.J. (2017). A novel fluorescent imaging technique for assessment of cerebral vasospasm after experimental subarachnoid hemorrhage. Sci. Rep..

